# Triphenyltin Ortho-Aminophenyl- and 2-Pyridyl-Thiolates: Synthesis and *In Vitro* Antitumour Activity

**DOI:** 10.1155/MBD.1996.75

**Published:** 1996

**Authors:** Marcel Gielen, Abdeslam Bouhdid, Edward R. T. Tiekink, Dick de Vos, Rudolph Willem

**Affiliations:** 1 Free University of Brussels V.U.B., Pleinlaan 2 Department of General and Organic Chemistry Faculty of Applied Sciences Brussels B-1050 Belgium; 2 Free University of Brussels V.U.B., Pleinlaan 2 High Resolution NMR Centre Brussels B-1050 Belgium; 3 Department of Chemistry The University of Adelaide Adelaide S. A. 5005 Australia; 4 Medical Department Pharmachemie BV Haarlem NL-2003 RN Netherlands Antilles

## Abstract

The synthesis, spectroscopic characterization and *in vitro* antitumour activity of two triorganotin compounds, triphenyltin ortho-aminophenylthiolate (**1**) and triphenyltin 2-pyridylthiolate, compound (**2**) are reported. The structure of **1** is confirmed by X-ray diffraction, with the tin atom in a distorted tetrahedral geometry because of monodentate coordination, as a thiolate (Sn-S 2.431(2) Å), of the ortho-aminophenylthiolate ligand. The *in vitro* antitumour activities of **1** and **2**, against a number of cell lines, are comparable to those exhibited by methotrexate and doxorubicin, and higher than those of carboplatin and cisplatin.


### TRIPHENYLTIN ORTHO-AMINOPHENYL- AND 2-PYRIDYL-THIOLATES: SYNTHESIS AND *IN VITRO* ANTITUMOUR ACTIVITY

Marcel Gielen[1a], Abdeslam Bouhdid[1a], Edward R. T. Tiekink[2], Dick de Vos[3], and Rudolph Willem[1a, 1b]

[1] Free University of Brussels V.U.B., Pleinlaan 2, B-1050 Brussels, Belgium
[a] Department of General and Organic Chemistry, Faculty of Applied Sciences
[b] High Resolution NMR Centre
[2] Department of Chemistry, The University of Adelaide, S. A. 5005, Australia
[3] Medical Department, Pharmachemie BV, NL-2003 RN Haarlem, The Netherlands

**Abstract**
The synthesis, spectroscopic characterization and *in vitro* antitumour activity of two triorganotin compounds, triphenyltin ortho-aminophenylthiolate (**1**) and triphenyltin 2-pyridylthiolate, compound (**2**) are reported. The structure of **1** is confirmed by X-ray diffraction, with the tin atom in a distorted tetrahedral geometry because of monodentate coordination, as a thiolate (Sn-S 2.431(2) Å), of the ortho-aminophenylthiolate ligand. The *in vitro* antitumour activities of **1** and **2**, against a number of cell lines, are comparable to those exhibited by methotrexate and doxorubicin, and higher than those of carboplatin and cisplatin.

**Introduction**
Diorganotin bis(2-pyridylthiolates) [1] exhibit an *in vitro* antitumour activity against MCF-7, a mammary tumour, and WiDr, a colon carcinoma ($ID_{50}$: 300 - 500 and 900 - 1300, respectively) comparable to that of cisplatin (850 and 624).
Because triphenyltin compounds are usually more active than di-n-butyltin derivatives [2], the most active of the diorganotin compounds, we synthesized triphenyltin ortho-aminophenyl- and 2-pyridyl-thiolates and screened their the antitumour properties *in vitro*.

**Results and Discussion**
*Syntheses*
Triphenyltin o-aminophenylthiolate, compound **1**, and triphenyltin 2-pyridylthiolate, compound **2**,

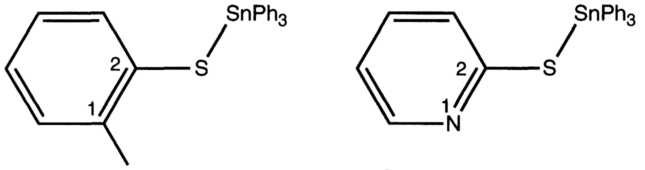

**1** **2**

were synthesized by the condensation of triphenyltin hydroxide with respectively ortho-aminothiophenol and 2-pyridylthiol.

*X-ray structure of compound 1*
Single crystals suitable for X-ray structure determination were obtained for compound **1**, but a full analysis for compound **2** was not obtained (see Experimental). The molecular structure of **1**, shown in Fig. 1, with the main interatomic parameters listed in the caption, is in agreement with a previous report [3]. The Sn atom exists in a distorted tetrahedral geometry defined by the three phenyl substituents and the S atom derived from a monodentate ortho-aminophenylthiolate ligand; Sn-S 2.431(2) Å. No evidence was found for a significant interaction between the Sn and N(1) atoms and this accounts for the relatively narrow range of angles about the Sn atom. The amino group forms two weak interactions with the S(1) atom, namely an *intra*molecular S(1)...H(1b) interaction of 2.58 Å (S(1)...N(1) is 3.018(4) Å) as well as an *inter*molecular S(1)...H(1a)' interaction of 2.84 Å (S(1)...N(1)' is 3.736(4) Å., symmetry operation: *x*, 0.5-*y*, -0.5+*z*). It is of interest to compare this structure with that of the dimethylated analogue [$Ph_3Sn(SC_6H_4NMe_2)$] [4] for which

Sn-S(1) is 2.429(1) Å and S(1)-C(2) is 1.774(5) Å (cf. 1.776(4) Å in the present study). In the latter structure there is a close *intra*molecular Sn...N contact of 2.893(3) Å which is responsible for the opening of a S(1)-Sn-C angle to 121.7(1)°.

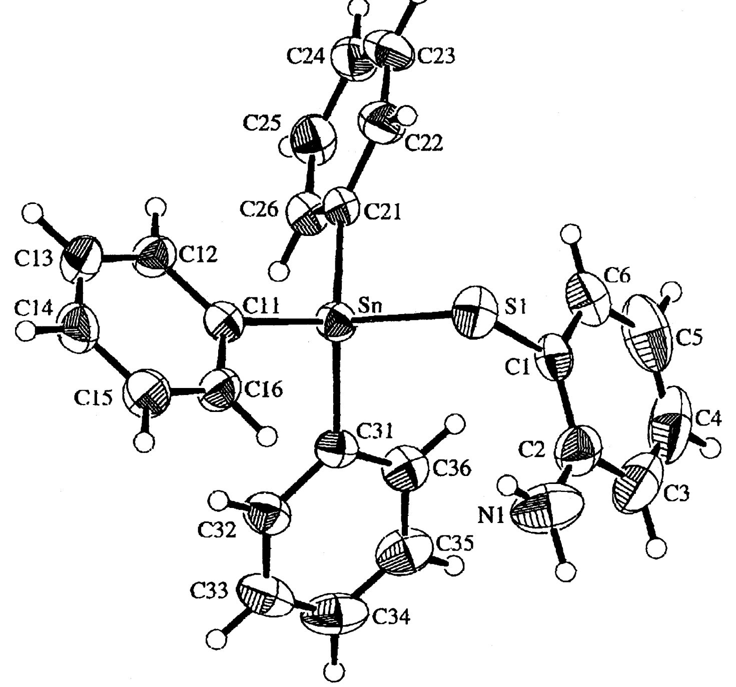

**Figure 1** Molecular structure and crystallographic numbering scheme for triphenyltin ortho-aminophenylthiolate, **1**. Selected interatomic parameters: Sn-S(1) 2.431(2), Sn-C(11) 2.133(4), Sn-C(21) 2.126(4), Sn-C(31) 2.134(3), S(1)-C(2) 1.776(4), N(1)-C(2) 1.343(6) Å; S(1)-Sn-C(11) 104.9(1), S(1)-Sn-C(21) 106.3(1), S(1)-Sn-C(31) 109.2(1), C(11)-Sn-C(21) 115.6(1), C(11)-Sn-C(31) 109.7(1), C(21)-Sn-C(31) 110.7(1), Sn-S(1)-C(1) 97.3(1)°.

Clearly, the orientation of the amino group away from the Sn atom in **1** occurs to facilitate the formation of hydrogen bonding contacts, a situation that can not occur for the dimethylated analogue. A full structure determination could not be achieved for **2** because of crystal disorder. The analysis confirmed nevertheless the generation of the desired compound and showed that the triorganotin center is coordinated by the S atom, leading to a distorted tetrahedral geometry; a weak *intra*molecular interaction with the pyridine-N atom is evident.

*Spectroscopic characterization of compounds **1** and **2***

The compounds were characterized by $^{1}H$, $^{13}C$, $^{119}Sn$ NMR and $^{119}Sn$ Mössbauer spectroscopy (see Experimental Section). $^{13}C$ assignments were achieved from the comparison of $^{13}C\{^{1}H\}$ standard and DEPT spectra, as well as from aromatic chemical shift increment calculations [6].

The $^{1}J(^{13}C-^{119/117}Sn)$ coupling constants (596/570 Hz) and the $^{119}Sn$ chemical shift (-116.3 ppm) of compound **2** suggest a tetrahedral monomeric structure, in agreement with previous NMR data [8]. For compound **1**, somewhat lower $^{1}J(^{13}C-^{119/117}Sn)$ coupling constants (558/533 Hz) are observed, but a quite different $^{119}Sn$ chemical shift (-68.0 ppm). These NMR data confirm the data from the solid state that the intramolecular N→Sn interaction is stronger in the 2-pyridylthiolate (**2**) than in the ortho-aminophenylthiolate (**1**). Thus in solution likewise both the low frequency shift of the $^{119}Sn$ chemical shift and the higher $^{1}J(^{13}C-^{119/117}Sn)$ coupling are indicative of a stronger distortion of the tetrahedral geometry towards five-coordination as a consequence of the weak N→Sn interaction. In contrast, the Mössbauer data, especially the very close QS values, do not discriminate between slight but significant differences in coordination spheres revealed by the NMR and X-ray data.

*Antitumour activities of compounds **1** and **2***

The *in vitro* antitumour activities of compounds **1** and **2** were determined against a panel of six human tumour cell lines, MCF-7 and EVSA-T, two breast cancers, WiDr, a colon cancer, IGROV, an ovarian cancer, M19 MEL, a melanoma, and A498, a renal cancer; the results are shown in Table 1.

**Table 1** *In vitro* antitumour activities (ng/mL) of compounds **1** and **2**, together with those of some reference compounds used clinically, against MCF-7 and EVSA-T, two breast cancers, WiDr, a colon cancer, IGROV, an ovarian cancer, M19 MEL, a melanoma, and A498, a renal cancer.

| Compounds | MCF-7 | EVSA-T | WiDr | IGROV | M19 MEL | A498 |
|---|---|---|---|---|---|---|
| **1** | <3 | 6 | 24 | 30 | 30 | 36 |
| **2** | 6 | 8 | 18 | 38 | 30 | 35 |
| Carboplatin | 10500 | 4500 | 3500 | 2400 | 5500 | 18000 |
| Cisplatin | 1400 | 920 | 1550 | 230 | 780 | 1200 |
| 5-Fluorouracil | 350 | 720 | 440 | 850 | 310 | 340 |
| Methotrexate | 15 | 26 | 7 | 20 | 18 | 16 |
| Doxorubicin | 25 | 13 | 18 | 150 | 21 | 55 |

The *in vitro* results show that the two compounds are as active or more active than methotrexate, and doxorubicin, and significantly more active than carboplatin, cisplatin and even 5-fluorouracil against all cell lines.

### Experimental

*Syntheses*

Compounds **1** and **2** were prepared by adding 5 mmole of triphenyltin hydroxide to a solution of 5 mmole of respectively ortho-aminothiophenol or 2-thiopyridine in 150 $cm^3$ toluene and 50 $cm^3$ ethanol. After refluxing for 6 h, distilling off the ternary azeotrope water/toluene/ethanol with a Dean-Stark funnel and half of the remaining solvent, the resulting mixture was cooled down to room temperature, filtered and evaporated under vacuum. The residue was recrystallized from chloroform/n-hexane.

*Characterization*

Mössbauer data: QS: quadrupole splitting; IS: isomer shift; $\Gamma_1$ and $\Gamma_2$: line widths, all in mm/s.
NMR data: all spectra were acquired from $CDCl_3$ solutions and referenced to the residual $C^1HCl_3$ resonance at 7.24 ppm for the $^1H$ spectrum, to the central $^{13}CDCl_3$ resonance at 77.0 ppm for the $^{13}C$ spectrum and to $\Xi(^{119}Sn)$ = 37.290665 for the $^{119}Sn$ spectra [5].
Abbreviations for coupling patterns: dd = doublet of doublets; ddd = doublet of doublets of doublets; m = complex pattern; b = broad; coupling constants are given in Hz in parentheses for $^nJ(^1H-^1H)$ for $^1H$ spectra. Other coupling constants are indicated explicitly. Calculated chemical shifts, using incremental rules [6], are given in parentheses.

Compound **1**: m.p.: 78-79°C; yield: 83 %; Mössbauer data: QS: 1.63; IS: 1.34; $\Gamma_1$: 0.85; $\Gamma_2$: 0.78; $^1H$ NMR data: 7.51 - 7.47, m: H(o); 7.40-7.29: H(m) + H(p); 7.19, dd (8, 1): H-3; 6.88, ddd (8, 8, 1): H-5; 6.48, dd (8, 1): H-6; 6.40, ddd (8, 8, 1): H-4; 4.08, b: $NH_2$;$^{13}C$ NMR data: C-2: 115.0 (117.5); C-1: 148.6 (147.6); C-6: 114.6 (116.3); C-5: 127.9 (126.3); C-4: 118.3 (119.8); C-3: 136.7 (130.5); C(i): 137.9 [$^1J(^{119/117}Sn-^{13}C)$ = 558/533]; C(o): 136.6 [$^2J(^{119/117}Sn-^{13}C) \approx 43$]; C(m): 128.7 [$^3J(^{119/117}Sn-^{13}C) \approx 57$]; C(p): 129.7; $^{119}Sn$ NMR data: -68.0.

Compound **2**: m.p.: 98-100°C; yield: 86 %; Mössbauer data: QS: 1.62; IS: 1.26; $\Gamma_1$: 0.78; $\Gamma_2$: 1.09; $^1H$ NMR data: 7.96, ddd (5, 1, 1): H-6; 7.77 - 7.63, m: H(o); 7.37 - 7.27: H-3 + H-4 + H(m) + H(p); 6.76, ddd (7, 5, 2): H-5; $^{13}C$ NMR data: C-2: 160.7 (151.8); C-3: 123.6 (124.9); C-4: 136.8 (136.5); C-5: 118.9 (121.0); C-6: 147.9 (150.0); C(i): 141.1 [$^1J(^{119/117}Sn-^{13}C)$ = 596/570]; C(o): 136.8 [$^2J(^{119/117}Sn-^{13}C) \approx 44$]; C(m): 128.7 [$^3J(^{119/117}Sn-^{13}C) \approx 60$]; C(p): 129.3; $^{119}Sn$ NMR data: -116.3.

*Instruments*

All NMR spectra were recorded on a Bruker AC250 instrument, using a QNP probe tuned at 250.13, 62.93 and 93.28 MHz for $^1H$, $^{13}C$ and $^{119}Sn$ nuclei, respectively.
Mössbauer spectra were obtained as described previously [8].
The *in vitro* antitumour screenings were performed as described earlier [9].

*Crystallography*

**1**: Intensity data for a colorless crystal (0.13 x 40 x 0.40 mm) of **1** were measured at room temperature on a Rigaku AFC6R diffractometer fitted with MoKα radiation, $\lambda$ = 0.71073 Å employing the $\omega$:2$\theta$ scan technique. A total of 4267 data ($\theta_{max}$ = 25.0°) were recorded and of these 3994 were unique of which 2945 absorption corrected [10] data satisfied the $I \geq 3.0s(I)$ criterion and were used in the subsequent analysis.

Crystal data for **1**: $C_{24}H_{21}NSSn$, M = 474.2, monoclinic, space group $P2_1/c$, $a$ = 13.11(1), $b$ = 19.04(1), $c$ = 9.071(8) Å, $\beta$ = 106.87(7)°, $V$ = 2166(6) Å$^3$, $Z$ = 4, $D_c$ = 1.454 g cm$^{-3}$, $F(000)$ = 952, $\mu$ = 12.83 cm$^{-1}$, transmission factors 0.867 to 1.073.

The structure was solved by direct methods [11] and refined by a full-matrix least squares procedure based on F [12]. Non-H atoms were refined with anisotropic thermal parameters and H atoms were included in the model at their calculated positions (N-H 0.95 Å and C-H 0.97 Å). At convergence $R$ = 0.027 and $R_w$ = 0.030 (sigma weights); the maximum peak in the final difference map was 0.28 eÅ$^{-3}$. The molecular structure is represented in Fig. 1 (drawn with ORTEP [13] at 35% probability ellipsoids). Supplementary material (fractional atomic coordinates, thermal parameters, all interatomic parameters and structure factor tables) are available on request from ERTT.

**2**: A total of five data sets for different samples of **2** were collected at both room temperature and at -70°C, however, only unsatisfactory analyses were obtained. Whereas the gross structural were determined ($R$ ca 12%), reliable geometric parameters were not; see text. Crystal data: monoclinic, space group $Cc$, $a$ = 17.179(5), $b$ = 15.256(1), $c$ = 8.142(5) Å, $\beta$ = 109.55(2)°, $V$ = 2010(1) Å$^3$, $Z$ = 4.

**Acknowledgments**

We thank Mrs. I. Verbruggen for the acquisition of the NMR data. We are also indebted to Mr. H. J. Kolker, Dr. J. Verweij, Prof. Dr. G. Stoter, and Dr. J. H. M. Schellens for the *in vitro* tests. This research was supported by the Belgian "Nationaal Fonds voor Wetenschappelijk Onderzoek" N.F.W.O. (grant number S2/5 CD F198, M.G.), by the Belgian "Nationale Loterij" (grant number 9.0006.93, R.W.), the Belgian "Fonds voor Kollecktief Fundamenteel Onderzoek" (grant number 9.0094.94, R.W.), the Human Capital and Mobility Programme of the European Community (Contract Nr ERBCHRXCT920016) and by the Australian Research Council.

**References**

1. Bouâlam, M.; Meunier-Piret, J.; Biesemans, M.; Willem, R.; Gielen, M. *Inorg. Chim. Acta* **1992**, *198-200*, 249

2. Gielen, M.; Lelieveld, P.; de Vos, D.; Willem, R. "Metal Complexes in Cancer Chemotherapy", Keppler, B. K., Ed.; VCH, Weinheim (1993), chapter 17, p. 383; Gielen, M.; Lelieveld, P.; de Vos, D.; Willem, R. "Metal-Based Antitumour Drugs", vol. 2, Gielen, M., Ed.; Freund Publ. House, Tel Aviv (1992), chapter 2, p. 29; Gielen, M.; El Khloufi, A.; Biesemans, M.; Mahieu B.; Willem, R. *Bull. Soc. Chim. Belg.* **1992**, *101* , 243; Gielen, M.; Meunier-Piret, J.; Biesemans, M.; Willem R.; El Khloufi, A. *Appl. Organomet. Chem.* **1992**, *6* , 59; Gielen, M.; Meunier-Piret, J.; Biesemans, M.; El Khloufi; A.; Willem, R. *Polyhedron*, **1992**, *11*, 1861; Gielen, M.; El Khloufi, A.; Biesemans M.; Willem, R. *Appl. Organomet. Chem.* **1993**, *7* , 119; Gielen, M.; El Khloufi, A.; Biesemans, M.; Kayser F.; Willem, R. *Appl. Organomet. Chem.* **1993**, *7*, 201; Gielen, M.; Biesemans, M.; El Khloufi, A.; Meunier-Piret J.; Willem, R. *J. Fluorine Chem.* **1993**, *64*, 279; Gielen, M.; Bouhdid, A.; Kayser, F.; Biesemans, M.; de Vos, D.; Mahieu, B.; Willem, R. *Appl. Organomet. Chem.*, **1995**, *9*, 251

3. Ng, S.W.; Kumar Das, V.G.; Lee, F.L; Gabe, E.J.; Smith, F.E. *Acta Crystallogr.*, **1989**, *C45*, 1294.

4. Kuz'mina, L.G.; Struchkov, Yu. T.; Rokhlina, E.M.; Peregudov, A.S.; Kravtsov, D.N. *Zh. Strukt. Khim.*, **1981**, *23*, 108.

5. Mason, J., Multinuclear NMR, Plenum Press, New York, **1987**, p. 627

6. Kalinowski, H. O.; Berger, S.; Braun, S. "Carbon-13 NMR spectroscopy", John Wiley, New York (1988).

7. Holecek, J; Nadvornik, M.; Handlir, K.; Lycka, A. *J. Organomet. Chem*, *241* (1983) 177; Nadvornik, M.; Holecek, J.; Handlir, K.; Lycka, A *J. Organomet. Chem*, *275* (1984) 43; Lycka, A.; Holecek, J.; Nadvornik, M.; Hadler, K. *J. Organomet. Chem*, *280* (1985) 323; Holecek, J.; Handlir, K.; Nadvornik, M.; Lycka, A *J. Organomet. Chem.*, *258* (1983), 147

8. Bouâlam, M.; Willem, R.; Biesemans, M.; Mahieu, B.; Meunier-Piret, J; Gielen, M. *Main Group Met. Chem.*, **1991**, *14*, 41

9. Kepers, Y. P.; Pizzao, P. E.; Peters, G. J.; Van Ark-Otte, J.; Winograd, B.; Pinedo, H. M. *Eur. J. Cancer*, **1991**, *27*, 897

10. Walker, N.; Stuart, D. *Acta Crystallogr.*, **1983**, *A39*, 158.

11. Sheldrick, G.M. SHELXS86, Program for the Automatic Solution of Crystal Structure, Göttingen, Germany, 1986.

12. teXsan, Structure Analysis Package, Molecular Structure Corporation, Texas, 1992.

13. Johnson, C.K. ORTEPII, Report 5136, Oak Ridge National Laboratory, Tennessee, 1976.

**Received: January 18, 1996 - Accepted: February 27, 1996 - Received in revised camera-ready format: March 1, 1996**

